# Real-Time Stereo-Based Ocean Surface Mapping for Robotic Floating Platforms: Concept and Methodology

**DOI:** 10.3390/s23083857

**Published:** 2023-04-10

**Authors:** Or Greenberg, Boaz Ben-Moshe

**Affiliations:** Kinematics and Computational Geometry lab., School of Computer Science, Ariel University, Ariel 4070000, Israel

**Keywords:** ocean surface mapping, real-time stereo vision, autonomous boat, wave detection and mapping

## Abstract

Consider the case of a small, unmanned boat that is performing an autonomous mission. Naturally, such a platform might need to approximate the ocean surface of its surroundings in real-time. Much like obstacle mapping in autonomous (off-road) rovers, an approximation of the ocean surface in a vessel’s surroundings in real-time can be used for improved control and optimized route planning. Unfortunately, such an approximation seems to require either expensive and heavy sensors or external logistics that are mostly not available for small or low-cost vessels. In this paper, we present a real-time method for detecting and tracking ocean waves around a floating object that is based on stereo vision sensors. Based on a large set of experiments, we conclude that the presented method allows reliable, real-time, and cost-effective ocean surface mapping suitable for small autonomous boats.

## 1. Introduction

This paper suggests a method for ocean surface mapping conducted from a floating object, such as a vessel, in real-time. In order to detect the waves and track their progress over time, experiments were performed with some spatial mapping sensors currently common in the autonomous tool industry, and from these sensors (i.e., Radar, LiDar, and stereo camera), the stereoscopic imaging system was selected as the leading sensor. The wave-mapping procedure determined through this study is intended for use in predicting the 6DoF (six degrees of freedom—location and orientation) state of the floating object in the near future for various uses in the autonomous tools industry. Unfortunately, such a prediction is extremely challenging due to the chaotic nature of the ocean surface, which is explained below.

The fundamental book *Chaos Theory Tamed*, by Garnett P. Williams, defines chaotic systems as ones that:

“… sustained and disorderly looking long term evolution that satisfies certain special mathematical criteria and that occur in a deterministic non-linear system”.

[1]

Simplifying this definition would label chaotic systems as ones that are extremely sensitive to minor and unpredictable, or unmodeled, changes in their initial parameters. As a result, those systems may be predictable in the short term but exhibit an unpredictable “random-like” behavior in the long term. This behavior is not due to actual random components that affect the system, as it is a deterministic system by definition, but rather from a high sensitivity to tiny changes in the initial conditions, which may develop a significant effect on the final behavior as they accumulate [2]. The ocean’s dynamics are a well-known example of a chaotic system [3]. Ocean surface movement is affected by various factors such as wind gusts, underwater currents, local changes in seabed depth, and more, which are all characterized as non-predictable components. It is not surprising, then, that predicting the spatial orientation (6DoF) of a floating object in the face of sea waves has been found to be not a simple challenge at all.

The methodology suggested by this paper involves combining continuous measurement of the vessel’s changing state over time using IMU components, with “look-ahead” measurements of the coming waves in order to evaluate the wave’s impact on the vessel and predict its 6DoF motion. Using the “look ahead” that detects and tracks the ocean waves before hitting the vessel, the random-like component of the sea surface’s behavior is reduced, and more reliable and accurate prediction of the vessel’s motion in the presence of the waves hitting it is made possible.

### 1.1. Motivation

Due to the chaotic nature of waves, they perform a major, yet unpredictable, influence on the deck’s immediate motion. The suggested methodology for ocean surface mapping using a stereo-based sensing system is intended to be used for predicting the future 6DoF state of a floating object in the near future, adjusted to autonomously controlled micro-size vessels (in terms of weight and cost). Such an ability can be used in a wide range of applications in the field of autonomous boats. One such research topic is optimized path planning (for autonomous boats). Such optimization may include energy consumption, modeling predictive control, minimizing tilt and turnover risks, or a combination of a few such objective functions [4,5,6,7].

Another possible application of such an ability lies in the field of conducting autonomous VTOL (Vertical Take-Off and Landing) missions on small floating objects [8]. The position and orientation of the vessel during the contact between it and the VTOL have enormous significance for the landing process, which has direct consequences for the success or failure of the operation. An error in the evaluation of the mutual orientation between the VTOL and the vessel at the moment of contact may lead to drift of the VTOL and an unplanned immersion in the cold ocean water. Moreover, even incorrectly estimating the rate of change of mutual orientation can cause fatal damage to both the VTOL and the vessel.

### 1.2. Related Works

This study deals with the detection and tracking of sea wave motion around a dynamic floating object, with the entire process being performed from the object alone. This prediction is intended to be used for predicting the 6DoF state of that object in the near future. Such a process includes a number of elements related to the dynamics of sea waves, wave-sensing technologies, and the deck’s state-prediction methods.

#### 1.2.1. Ocean Dynamics

**Figure 1:**
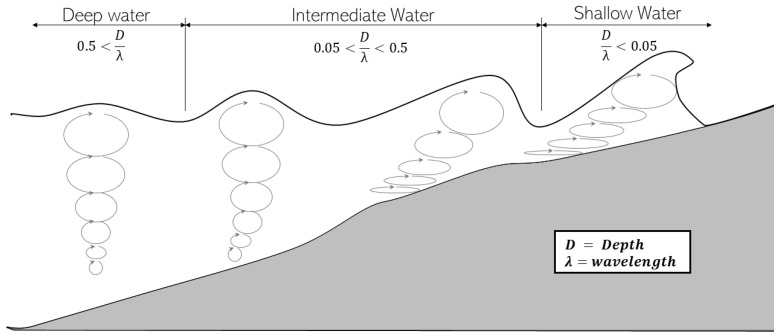
Deep and shallow water waves.

Generally speaking, waves are oscillations (or disturbances) of the water surface that can be observed in any water basin, such as rivers, lakes, seas, and oceans [9]. The dynamics of different types of waves are at the heart of the literature, and new studies in this field are published daily. Although diving into the dynamics of the ocean is out of the scope of this paper, a simple understanding of the basic phenomena is important. J.J. Stoker 2011 analyzes the mathematical and physical theory for the formation of different ocean waves [10]. Among other things, Stoker refers to the differences between deep-sea waves and shore waves. Research has already been distinguishing between deep-water waves and shallow-water waves for decades (see Morison et al., 1953 [11] and others), where “deep water waves” waves are defined as ones that are unaffected by the sea floor. In particular, when the depth is much larger than the wavelength (i.e., $\frac{D}{\lambda }>1/2$, where *D* is the water depth and λ is the wavelength), the wave-induced water particle motion weakens with depth and eventually vanishes at a depth approximately equivalent to half the wavelength ([12,13]). On the other hand, if the water depth is much smaller than the wavelength (i.e., $\frac{D}{\lambda }<1/20$), the water column is insufficient to allow complete development of the wave motion, affecting the properties of the surface oscillation. Waves are thus defined as shallow-water waves. For water depths between these two conditions, waves are only partly affected by the bottom topography, and waves are defined as intermediate-water waves. Figure 1 demonstrates the structural difference between shallow-water waves and deep-water ones

As demonstrated in Figure 1, waves coming onto the beach increase in height and steepness and eventually break [14]. The implication of this observation for our purpose is that deep-water waves are usually lower and less “pronounced” than shallow-water waves. Another significance of this observation, which is explicitly articulated by Stanislaw R. Massel [15], is the fact that deep-ocean waves vary slowly. This observation is critical for the present study that suggests using early detection of a sea wave to estimate the future motion of a vessel expected to meet that very same wave in the near future. In addition, while it is a matter of common observation that the ocean surface structure changes rapidly at the Macro-Level, we have shown that rapid changes take place at the Micro-Level also, where feature-points vanish rapidly (see Section 3 below), making the environment’s structure extremely dynamic and amorphous.

#### 1.2.2. Ocean Surface Mapping

A few wave-measuring methods have been developed over the years, from traditional surface-following buoys [16] to innovative satellite-based measurements [17]. Mori, Yasukuni et al. [18], and Westfeld, Patrick et al. [19] suggested LiDAR as the leading sensor for ocean surface mapping, while Kusters, J.G. et al. [20], Cui Jian et al. [21], and others suggested using different RADAR systems for that purpose. A third option, which is discussed in detail below, is stereo vision. A few previous researchers tried to use stereo vision in order to achieve a 3D ocean surface mapping and wave detector. Very good results were achieved by Corgnati Lorenzo et al. [22] in near-shore wave detecting and tracking using stereo vision. However, the proposed model was constructed for detecting nearshore waves in the coastal environment, which differ substantially from deep-ocean waves in both physical structure and dynamics.

One remarkable project in the field of ocean surface mapping using stereo is WASS (Waves Acquisition Stereo System) [23]. This work provides high-quality 3D point-cloud recovery from stereo frames of the ocean surface. Yet the whole process is not applied in real-time (30 s per frame for 3MPixel images on a consumer i7 CPU according to documentation). In addition, WASS provides high-quality results for stereo shots taken from a high angle with respect to the water surface. Yet experimental production of point-clouds for stereo shots taken from low angles showed massive degradation in the product’s quality compared to the high-angle case.

#### 1.2.3. Deck’s 6DoF Estimation for *VTOL* Applications

As mentioned, one important motivation for this research lies in the field of *VTOL* on vessels. Previous research intended to develop capability for VTOL applications on vessels. One early product, published in 2016 [24], suggested a multispectral sensor-fusion system that managed to estimate the deck’s state for UAV landing applications. This system offers a Bayesian filter-based algorithm for estimating the current boat’s state based on an electronic platform-model prediction unit (according to a predetermined platform motion model) and measurements from a multispectral sensor-fusion system (including LiDAR, RADAR, VSI, and IR) located on the aircraft. Another paper [25] uses an MSS hydro toolbox to simulate a ship’s 6DoF motion, then the ship’s Heave, Pitch, and Roll motions are predicted for 5 s after 100 s without measurements using the NARX network. In addition, a few more researchers (such as Lim Edward [26], Elgersma Michael Ray et al. [27], and others) have suggested different methods with the more general goal of landing an aircraft on a vessel. The majority of these methods do not focus on the deck’s state computation or prediction but on the landing process itself (autonomously or manually).

### 1.3. Our Contribution

Mapping the ocean surface is commonly performed using marine radar systems (e.g., in [28]; more examples are reviewed in [29]). Such systems are relatively expensive and have considerable power consumption (required for RF chirp transmission). In this paper, we present a real-time method for performing ocean surface mapping based on stereo vision that is affordable, widely available (Commercial Off-The-Shelf—COTS), and suitable for small and micro autonomous boats (often named Autonomous Surface Vehicles—ASVs).

Existing projects in the field of ocean surface mapping and wave detection (e.g., the ones presented above) lacked the combined ability to perform in real-time and from relatively low shooting angles and to adequately detect deep-ocean waves rather than near-shore ones. The marine environment has no “static features”. Therefore, computer vision methods that are based on detecting “feature points” over time (such as structure-from-motion, optical-flow, and SLAM) tend to perform poorly on such dynamic scenes. To the best of our knowledge, this is the first real-time wave-mapping method using stereo-vision and is applicable for very small (toy-graded) unmanned autonomous boats.

### 1.4. Paper Structure

This paper is organized as follows: Section 2 presents the preliminary properties of ocean waves and available sensors for mapping such a surface (mainly focusing on stereo vision sensors). Section 3 extends the stereo-mapping process to be a continuous process in time. In Section 4, the presented framework is tested in field experiments both in the open ocean and a controlled environment. Finally, in Section 6, we present several generalizations and applications for the suggested wave-mapping method and conclude the paper with suggestions for possible future work.

## 2. Wave Sensing

Our aim, therefore, is to describe a methodology for mapping the sea surface, with an emphasis on wave detection.

The task of ocean surface mapping and wave detecting entails a variety of challenges. First, both the mapped scene and the mapping system (i.e., the sea surface and the vessel, respectively) are in continuous motion. That is, the geometry between the mapping system and the world-space frequently changes. Moreover, not only does the ocean surface change its position over time, but its shape also changes rapidly. i.e., the exact same wave may appear completely different after a relatively minor amount of time. Therefore, we strive to produce a system capable of sampling at a rate not less than 10 Hz.

The distinction between wave detection and obstacle detection should be noted. Obstacle detection primarily involves identifying, tracking, and locating rigid objects that exist either above or below the sea surface, such as rocks or swimmers. In contrast, waves are not considered rigid objects, and therefore a given pixel cannot be classified as “wave” or “no-wave” on a binary scale, which is possible with rigid obstacles. The nature of waves as a continuous, non-discrete phenomenon makes their detection substantially different from that of rigid objects.

### 2.1. Sensor Review

**Figure 2:**
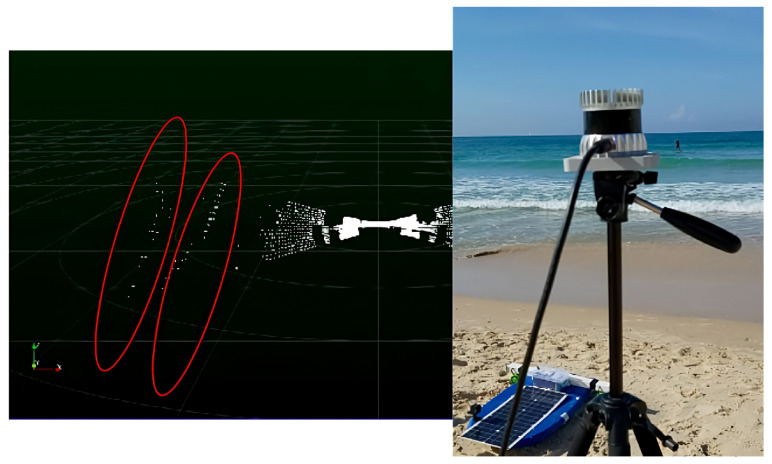
Shore LiDAR setup: two minor shore waves are easily detectable—marked by red ovals.

**Figure 3:**
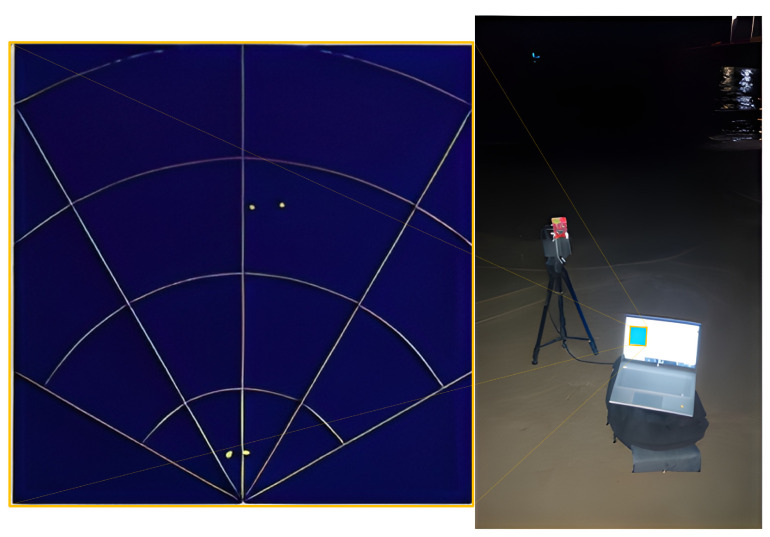
The mm-wave radar setup during a shore experiment.

For that task, we tried using spatial mapping sensors commonly in use in the autonomous vehicle industry, in particular, multi-ray long-range LiDARs, mm-wave RADARs (see Figure 2 and Figure 3 respectively), and stereo vision. It should be noted that the focus of this study is on relatively small vessels (up to several hundred kilograms), and therefore cost considerations were a significant factor in choosing the preferred sensor.

Due to a combination of considerations arising from the sensors’ performance in preliminary experiments (detailed below), cost and weight considerations, as well as part of the global trend of increasing the use of vision-based sensors in the autonomous tools industry, it was decided to use a stereoscopic imaging system as a leading sensor for the current study.

### 2.2. Failed Attempts

While LiDAR wave sensors perform relatively well inshore, for deep-ocean waves they simply fail to detect the ocean surface. We attribute this failure to the natural penetration property of water, combined with the low reflective nature of water. Even high-level LiDAR (best in class: 240 m, 128 rays) simply failed to detect the reflected laser signals in deep water.

While testing different RADAR systems, we ran into some different problems. Since most of the existing products are meant to focus on rigid objects, the ever-changing water surface seemed to the system as noises to be filtered in order to avoid a noisy RADAR image that interrupts the rigid components on which the system is tuned to focus.

### 2.3. Introduction to Stereo Vision

Stereo vision is a vision-based technique using multiple cameras (at least two) filming the same scene from different views to extract 3D information, commonly used in the robotics industry (e.g., [30,31,32] and others). The basics of this technique are pretty intuitive and can be easily demonstrated by the “finger model” as follows. Place your finger vertically right in front of your eyes, about 5 cm away from your nose, and look at it with only one eye open. Now, switch eyes. If you repeat this process a few times rapidly, you will surely see that the finger “changes position” between the two views (the use of quotes is because the finger is clearly not changing its position, but the different point of view causes this illusion). Now, repeat this process one more time, but now locate your finger farther away (30 cm away from your nose for that matter). You may notice that even though the finger is still “changing position” between the views, the changes are much more minor than in the previous setting. This “position changing” of objects between two views (i.e., two images) is the heart of the stereo-vision technique. By comparing the locations of two corresponding points in these two images, relative depth information can be obtained in the form of a 3D point-cloud or a disparity map, encoding the difference in coordinates of each two corresponding points to a three-dimensional coordinate.

**Figure 4:**
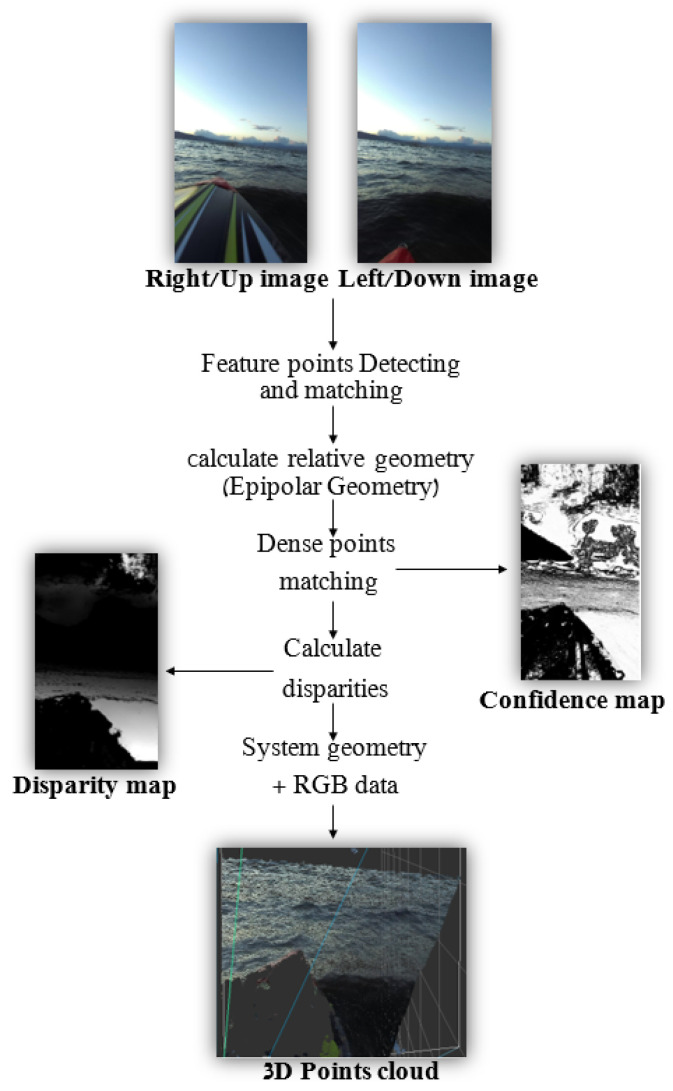
An overview of a Dense 3D reconstruction framework using stereo-vision.

For the purpose of this paper, we assume that the reader has at least a basic prior knowledge of stereo vision, and in particular is familiar with the basic concepts in stereo calculations and Epipolar Geometry such as Epipolar Lines, Fundamental and Essential Matrices, and Disparity Calculation. The overall framework of 3D reconstruction from a pair of images using stereo-vision (Dense) is described in Figure 4.

As presented in Figure 4, the very first step of the stereo calculation process is detecting and matching corresponding points within the two given images. These points are used to evaluate the relative geometry (also known as Epipolar Geometry) between the two views [33]. Epipolar Geometry enables an efficient search of a matching pixel from Image B for each pixel from Image A. Without getting too deep into details, Sparse stereo matching finds many “feature points” in one image (using classic algorithms such as Harris [34], SIFT [35], SURF [36], etc., or more-innovative methodologies such as R2D2 [37] or DELF [38]), then uses the Epipolar Geometry to find the “best” corresponding points on the other one, whereas Dense stereo matching goes over “all” the pixels in one image and finds the “best” match for each one of them. Once we have pairs of corresponding pixels, we can calculate the disparities for each pair and build a disparity map. This map can be translated to a depth map or a 3D point-cloud using the system’s parameters—focal length and baseline.

The term “best match” holds a significant hidden parameter characterizing each pair—the confidence level. In order to define the confidence level parameter, a short introduction to point matching should be given. In fact, we do not match a single pixel from one image to another single pixel from the other one, but we match templates. A template is a patch of n×m pixels from Image A, and the purpose of matching is to find a patch in Image B that is the most similar to it. Epipolar Geometry allows reducing the region of interest from finding the corresponding patch from the whole image to the Epipolar-line corresponding to the point that defines the current patch.

The basic matching algorithm is simple—given two images with a known Epipolar Geometry, apply the following:

(i) For the required pixel [i, j] in image A, define a template as an n×m patch containing the pixel [i, j] (the patch will usually be defined such that [i, j] will be in its middle).
(ii) Calculate the Epipolar line corresponding to [i, j] using the Fundamental Matrix.
(iii) For every n×m patch along the Epipolar line in Image B, evaluate the similarity of that patch to the template.
(iv) Choose the middle pixel [$i^{\prime },\;j^{\prime }$] of the most-similar patch as the “best match” and repeat the process with the next required pixel.

Now, we may define a confidence value for each matched pair as the level of distinctness of the “best” patch chosen, i.e., how much are we sure that the pixel we chose from Image B is actually the one corresponding to the pixel from Image A.

### 2.4. Stereo Adjustment to Wave Detecting

Seemingly, stereo vision is not the ideal candidate for ocean surface mapping. On the one hand, previous studies (such as the previously discussed WASS project) have shown that a pair of cameras do have the needed sensitivity for the mission under some limitations. On the other hand, these limitations may be critical for our needs. In order to perform effective wave detection for the deck’s 6DoF state-evaluation application, the system has to perform from the vessel only, i.e., from a relatively low shooting angle, and in real-time.

**Figure 5:**
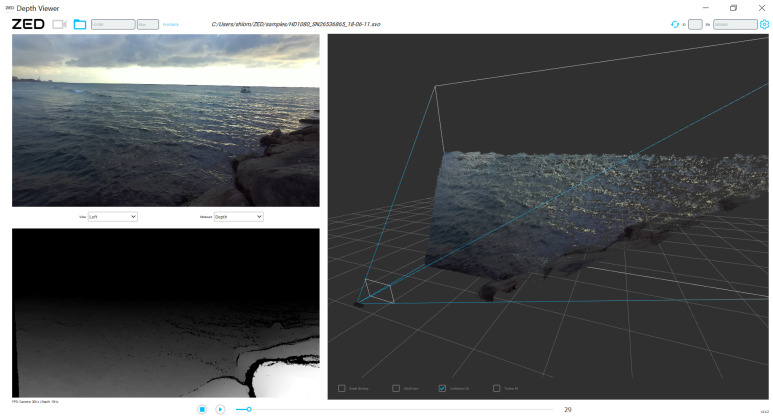
Example 3D ocean surface stereo-based mapping using a ZED2 located on a pier 2.5 m above the surface. The (**top left**) image, (**bottom left**) disparity map, and (**right**) 3D point-cloud are presented.

For this study, we mostly used stereo cameras from the ZED series (in particular *ZED* 2 and *ZED* 2i, SDK version: 3.4.2.) produced by StereoLABS, which are offered as off-the-shelf products and are widely used in the autonomous tools industry. These cameras allow stereoscopic mapping over an effective range of approximately 15–20 m (with a 12 cm baseline) and a choice between different resolutions and frame rates. Activating the camera using NVIDIA’s Jetson Xavier NX computing platform enabled real-time mapping of the sea surface from a high angle in 1080p (full-HD) resolution at 30 fps (frame per second); see Figure 5.

As described above, our goal is to show that effective sea wave detection can be performed in real-time and from a low angle using stereo vision. Considering these goals, this initial experiment drew two substantive observations. The first one is positive—it seems that the computation time challenge can be solved relatively simply by using the appropriate hardware to obtain real-time estimation. However, the second observation seems to pose a more significant challenge. A close look at Figure 5 shows that the farther the mapped area is from the baseline, the more areas of uncertainty tend to appear. These areas are expressed as a discontinuity in the 3D point-cloud or “black holes” in the disparity map (see the far-right part of Figure 5). This observation is significant as it implies that stereoscopic mapping becomes less effective the sharper the shooting angle is with respect to the sea surface. Being aimed to perform from relatively small vessels, this observation is of major significance.

An even closer look at this phenomenon provides an interesting diagnosis as to the origin of the emergence of these areas of uncertainty. Comparison between the upper-right part of the stereo product in Figure 5 and the upper-left part of it shows that these “holes” do not appear as a direct derivative of the distance from the system’s origins, but rather, as the angle between the base and the photographed object becomes sharper, the calculation becomes more sensitive to occlusions. In fact, due to the direction in which the camera in Figure 5 is pointed with respect to the direction of the sea surface’s motion, the far-left part of the product is photographed with a side view perpendicular to the waves advancing. That way, almost no occlusions are formed; so in this area, the product remains continuous even at long distances. In contrast, on the far-right side of the product, the photographs are taken with a frontal view relative to the direction of the advancing waves. As a result, more occlusions are created, which cause areas of uncertainty over long distances (i.e., low angles). This diagnosis leads us to an interesting understanding—instead of focusing on what the system is able to see, the occlusions can be used to try to emphasize what the system is unable to see. Knowing that the cause of these occlusions is waves, early detection of these areas of uncertainty can provide a reliable measure of the presence of a wave in the distance making its way toward the boat.

Focusing on occlusion detection using the stereo product to evaluate the presence of sea waves, we decided to examine two stereo setups: a horizontal mode and a vertical one. In the horizontal setup, the system is positioned so that the two cameras are displaced in the horizontal direction (in a manner that maintains a right camera and a left camera located at the same height above the water surface), while in the vertical setup, the cameras are spaced apart in the vertical direction.

The most commonly used stereo system, the human eyes, are horizontally displaced from each other, leading to the traditional use of horizontally displaced stereo photography systems. The motivation for examining the vertical setting (as opposed to the trivial horizontal setting) is twofold. First, the horizontal setting, similar to human vision, is aimed at identifying vertical objects such as other people, trees, buildings, and so on. Since the depth estimation method using stereo is based on detecting disparities, i.e., the object’s displacement between the two views, vertical objects’ disparities are more easily detectable along the horizontal axis. A good demonstration of this attribute can be observed using the previously presented “finger model”. Repeating this demonstration while placing the finger horizontally in front of the eyes, and not vertically as suggested in the first place, shows that the differences between the views are more noticeable when setting the finger vertically rather than horizontally. Conversely, in view of waves’ typically horizontally structured elements, it can be assumed that a vertical setup of the system can make detection of disparities more efficient. Second, focusing on occlusion detection, we assume that shifting the cameras’ origins apart along the vertical axis may highlight the occlusions resulting from areas visible to the higher camera but hidden from the lower one.

In addition, since we are aiming to find areas of uncertainty, we would like to recover the confidence map and search for low-confidence areas that can hint at the presence of the wave that created them. As described above, the confidence map is a description that encodes the confidence level of the evaluated location of each pixel in the dense disparity map, calculated using Epipolar Geometry. Since the wave heads “hide” wider areas from the lower camera than from the upper one, we anticipated that wave detection could be easily done using the confidence map, with an emphasis on placing the stereo in a vertical setup. Finally, we would like to use the stereo product as an input for a real-time wave detector. The Hough Transform is an algorithm patented by Paul V. C. Hough [39] then perfected by Duda and Hart [40] to recognize complex lines in photographs. In view of the waves’ typical line structure, we would like to suggest the Hough transform as a naive and simple tool for wave detection from a stereo product.

## 3. Stereo-Based Wave Tracker

**Figure 6:**
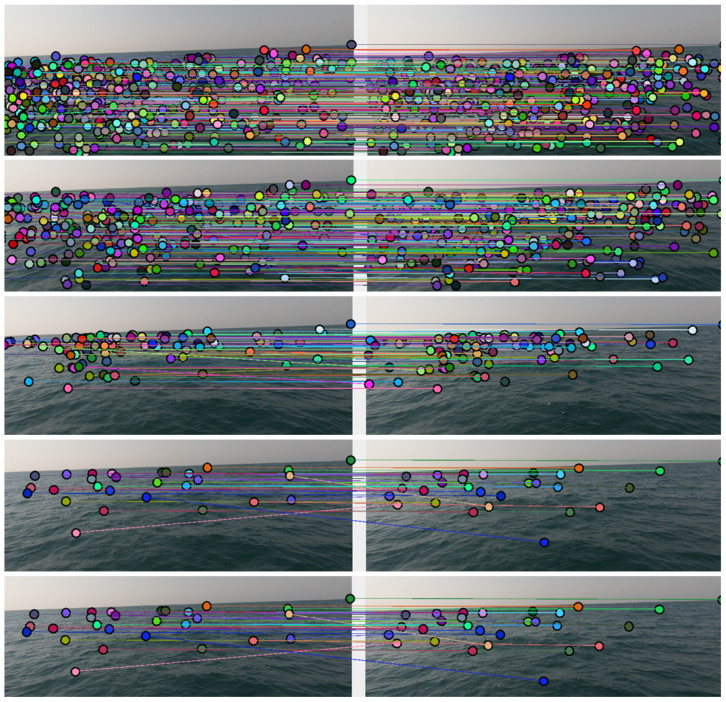
Matches between two consecutive frames of the very same scene at different rates: 120, 60, 30, 15, and 7.5 fps (from top to bottom). Originally recorded with a base filming rate of 120 fps in 1080p (full-HD) resolution.

After establishing a stereo-based wave detector, we would like to extend the problem in the time domain. In general, vision-based deep-water wave tracking is not a trivial mission. The significant challenge of tracking wave motion using “traditional” feature-based tracking tools lies in the amorphous and ever-changing nature of the sea surface. While rigid objects (people, cats, vehicles, etc.) maintain an implicit assumption that the shape changes they undergo between a pair of consecutive frames is small to negligible, this assumption is not at all straightforward when it comes to sea waves. Sea waves unite, split, and change their observed shape rapidly. The same wave can look completely different in two frames where the time difference is only a small fraction of a second. As a result, feature-based tracking has been proven to be completely unreliable, especially when using relatively low filming rates. Figure 6 depicts attempts to match feature points between a pair of consecutive frames of the same scene filmed at different filming rates.

It is noticeable that as the filming rate decreases, fewer and fewer matches are successfully made between consecutive frames, and those that are made include increasing matching errors. In particular, feature-matching at 60–120 fps was able to produce a large number of matching pairs at different shooting ranges. However, feature-matching at lower frame-rates resulted in a particularly small amount of matches and many matching errors. Yet although feature-based wave motion tracking is a particularly challenging task, it seems stereoscopic mapping can be harnessed to our aid here, too. Using the disparity map allows changing the methodology from feature-based tracking to an object-based one. In addition, the transformation from RGB to a disparity or confidence map performs a dimensionality reduction. While the RGB image preserves the detailed shape of the wave with high precision, the stereo product preserves only a sub-sampled version of the wave. That way, operating fairly standard object-tracking algorithms on the disparity map (using the waves detected by the previously discussed Hough detector) allows for accurate tracking of sea waves at a sampling rate not exceeding 10 fps and can be performed in real-time. It can also be assumed that the trend of the progress of waves at sea depth is relatively constant (i.e., does not change rapidly or sharply). This assumption allows for refining the tracking using simple Bayesian tracking algorithms (such as LKF).

Tracking wave motion in time should be used to estimate the average wave velocity, and hence the time to impact. Unfortunately, it turns out that estimating the wave motion by simply calculating the difference between its image coordinates in time *t* and time t+Δt does not do the job. Since the ship from which the mapping is performed may also possibly be in rough motion throughout the mapping process, calculating the absolute differences in the wave’s coordinates between two consecutive frames of the stereo product is not sufficient to assess the wave’s progress. That is due to the fact that the displacement in coordinates between the two frames may stem not only from the wave’s progress but also from the ship’s changing orientation. Using an IMU device for measuring the ship’s motion, a three-dimensional transformation of the system between any two consecutive frames may be a solution to this problem. However, such a transformation requires significant computational resources and may add error factors resulting from miscalculating the conversion parameters or due to numerical issues. We want to offer a simpler solution. In fact, we want to isolate the displacement component resulting from the actual wave progress from the total displacement in its coordinates between two consecutive frames. For this purpose, we want to create a reference point whose location is not fixed in the image-space, but moves in a way that embodies the ship’s movement. This way, the relative distance between the detected wave and the reference point embodies the wave progress only, with no side effects of the ship’s motion.

**Figure 7:**
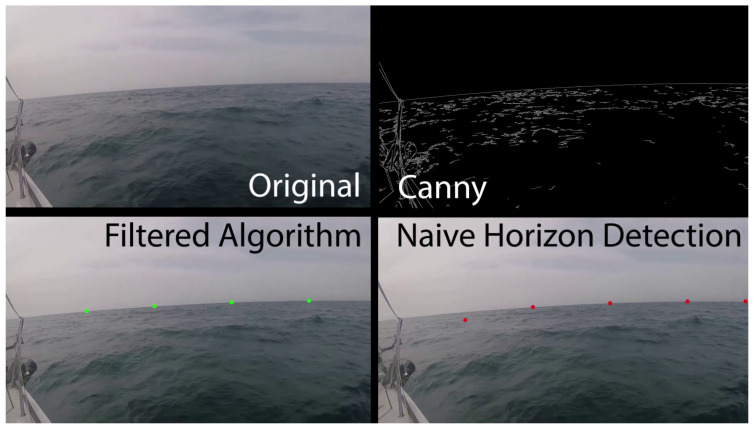
Horizon line detection algorithm. Image from https://www.youtube.com/watch?v=ywHjs-sOBhY (accessed on 5 April 2023) “Horizon Line Detection Algorithm” by Dr. Roi Yozevitch.

A good candidate to function as such a reference point is the horizon line. Being a fixed static element in “world” coordinates, the horizon line’s motion in video photography (as a good enough approximation) results from the ship’s motion alone. In addition, the horizon is a prominent object in any frame and can be identified relatively easily from a single camera shot. The suggested horizon line detection algorithm is relatively simple and is demonstrated in Figure 7 below.

This is a three-stage algorithm that receives video as input and returns n points (default—four points) spanning the horizon line as output, as follows:

(i) For each frame in the input video, we first run a Canny algorithm to detect edges within the image.
(ii) We “naively” look for the points that span the horizon. A suggested option to perform this operation is to fix the width coordinate of n points in as $\frac{w\times i}{n}$, where *i*[0→(n−1)] denotes the point’s index, *n* the total amount of points, and *w* the image’s total width in pixels. Now, we scan the vertical lines in the edge image defined by the width coordinate of each point and look for the first height coordinate where the line meets a pixel value that is not “black”. The working assumption is that in the general case there are not likely to be extreme edges in the part of the image indicating “sky”, certainly compared to the part of the image indicating “water”. Since the horizon line is a continuous and most-prominent edge in the image space, the algorithm is expected to detect it with high accuracy.
(iii) We now refine our assessment of the horizon line’s position. We perform one local refining for the location of each point individually using a Bayesian filter for a “random noise” motion model in time, and a second general refining for filtering points inconsistent with the characteristic of the almost linear shape of the horizon line.

The described algorithm provides accurate real-time detection and tracking of the horizon line. A qualitative estimate of the wave’s progress can now be obtained by measuring the change in distance between the wave’s position and the position of the horizon line between consecutive frames. Combining this tool for time-to-impact estimation with the previously discussed tool for wave detection and estimation provides a high-quality snapshot that can serve as a basis for predicting the ship’s future 6DoF state.

It should be noted that the proposed vision-based horizon-detection algorithm was designed originally to allow the use of stereo cameras without an IMU. Yet after several attempts we were able to properly synchronize the images from the stereo cameras with the IMU sensor readings. Therefore, the suggested vision-based horizon detection was mainly used to calibrate the stereo camera with the IMU and not as a continuous orientation sensor.

### System Overview

**Figure 8:**
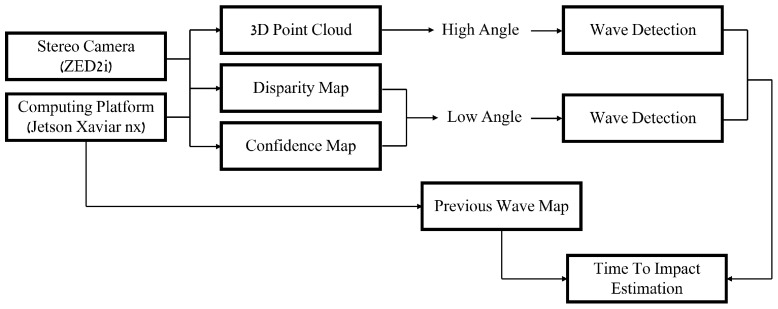
A block diagram of the suggested approach.

This section presents a block diagram of the proposed approach and summarizes the system’s overall framework. The block diagram is presented in Figure 8.

The system requires visual input provided by a stereoscopic imaging system. The imaging system includes at least two cameras displaced from each other in either a vertical or a horizontal manner. We showed that a vertically displaced system provides more-accurate results over long ranges, while a horizontally displaced one is able to perform better for short ranges. While over short ranges (i.e., high shooting angle with respect to the sea surface) wave detection can be performed relatively trivially using the 3D point-cloud, over long ranges (i.e., low angles), we use the confidence and the disparity map to detect occlusions derived from the presence of waves. Finally, the previous wave map is compared to the current one to estimate the wave’s progress in time and to evaluate the time-to-impact.

## 4. Experimental Results

During the research process, the system was tested under various conditions and environments. As a vision-based technique that may suffer from occlusions, saturation, glare, lens flare, and other environment-based interruptions, it was decided to establish the experimental approach on field experiments over simulated ones to get actual representative data.

**Figure 9:**
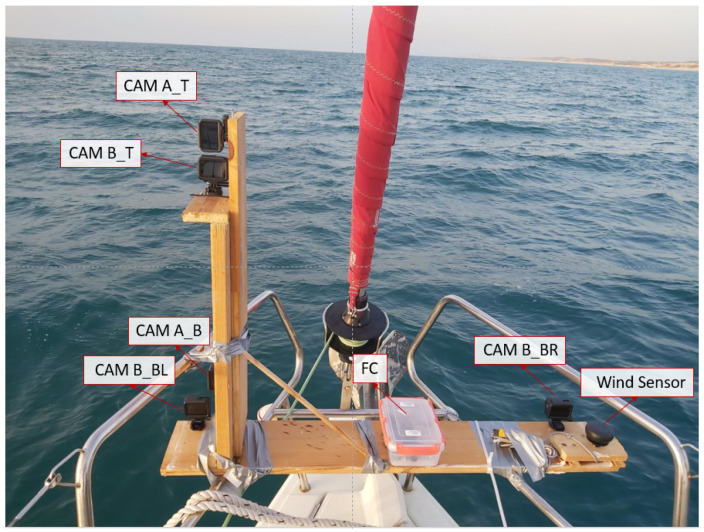
Multiple-camera wide-baseline stereo system located on an Elan 40 yacht during a field experiment. The stereo system is based on Go Pro cameras (Hero Black 7) and consists of 2 camera arrays: (*CAM-A*) two cameras, vertically displaced from each other. (*CAM-B*) 3 L-shaped cameras. Additional sensors: FC and ultrasonic wind sensor for future applications.

As mentioned, stereo-based mapping depends directly on the ability to match feature points between two images. These feature points are used to understand the relative geometry between the two image planes to produce a dense disparity map based on the Epipolar Geometry (see Figure 4 above). In addition, the range of efficiency of the stereoscopic system is directly related to the baseline length, i.e., the distance between the two cameras’ origins. In fact, most stereoscopic systems currently offered for purchase as off-the-shelf products have a relatively short baseline of about 7–10 cm and are therefore limited to an effective sensing range of about 15–20 m at most. In order to use stereo-based mapping with long-range capabilities, a wide-baseline system should be used. For this purpose, we assembled a homemade stereoscopic photography system based on GoPro cameras (Hero Black 7) separated from each other at a 1.1 m baseline. This system included five cameras performing in two independent stereo systems installed on the front deck of an Elan 40 RACER CRUIZER yacht (see Figure 9). As part of the experiment, synchronized stereo images of the deep-sea surface were captured 1–3 km off-shore in the Middle Sea (the Mediterranean Sea). The recordings were taken at different resolutions (from 720p to 4K) and in low-wave conditions (up to 30 cm).

**Figure 10:**
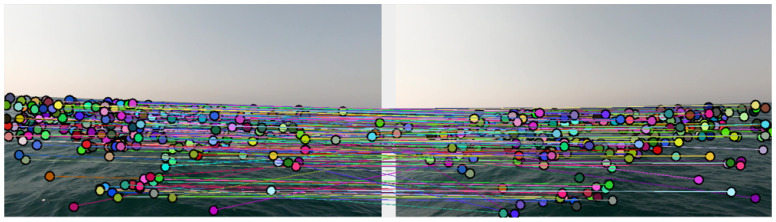
Feature-point matches on the ocean surface from 1.1 m baseline stereo images.

In order to prove the feasibility of mapping the sea surface using a stereo system with a wide baseline, we would like to show that detecting and matching of feature-points on the sea surface between a pair of images taken from such a system is possible even at long distances. For this task, several platforms were examined, and the BoofCV [41] library by Peter Abeles was selected for its relatively good performance compared to similar platforms. Although the platform was able to find and match a relatively large number of points between the two given images (see Figure 10), plenty of matching errors were observed, making stereo-evaluation impossible over these matches.

Some of the “bad” matches can be pre-filtered according to an extreme criterion—rough matching errors that are expressed in an extreme angle of the line connecting the two points when placing the two images side-by-side (examples of such errors can be seen in Figure 10). Yet this rough filtering process can not detect the “subtler” matching errors, which require a more fine-filtering methodology. For this purpose, we considered the basic linear pattern of the sea surface. In contrast to urban environments where buildings, pillars, signs, etc., frequently interrupt the basic structure of the plain, on the sea surface the situation is different. Photographing the sea surface using a single camera, the plane is deployed almost linearly in the sense that as a photographed object is farther in world space, the distance is expressed by the “rise” of the feature point representing this object along the vertical axis of the image plane. The stereo model teaches us that given a pair of corresponding points matched between two views of the same scene, the parallax, or disparity, between the two points is inversely related to the distance in the world between the system’s baseline and the object they represent. If so, it can be expected that the ratio between the position of the point in the height axis of the mono-image and the parallax of that point from the corresponding point in the second image is linear or close to linear.

**Figure 11:**
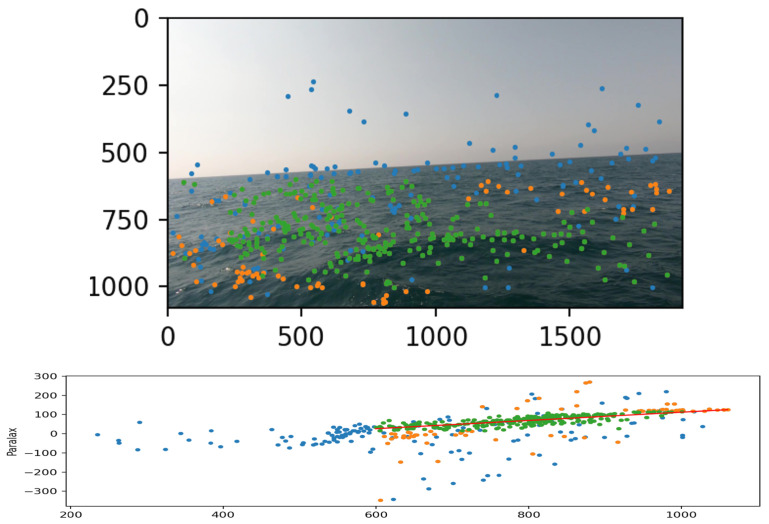
Matches filtered by extreme criterion (blue) and linearity criterion (orange). Final matches in green.

Figure 11 shows the product of the proposed filtering algorithm according to the two suggested criteria—the extreme criterion (combined with filtering points above the horizon line) and the linear criterion. It can be seen that, indeed, most of the matches maintained behavior close to the expected linear behavior, and those that did not do so could be effectively identified and filtered. It seems that identifying and matching feature points between a pair of sea surface images taken using a wide-baseline stereoscopic camera system does not constitute an algorithmic challenge relative to existing short-baseline stereoscopic systems, and that the challenge of producing such a stereo is primarily an engineering challenge not discussed in this paper. This article discusses the use of stereoscopic mapping for the purpose of detecting waves and predicting a deck’s orientation using existing stereoscopic systems with a short baseline, where the suggested methodology offers the future possibility to expand the mapping range by increasing the system’s baseline.

Although the homemade system has shown the feasibility of stereo production in terms of feature-point matching, it turned out that it lacked some more technical support in terms of stabilization, calibration, etc. In addition, the Middle Sea did not provide high waves with respect to the ship’s height, which did not challenge the system in terms of occlusions. As a result, it was decided to down-scale the scenario and use a short baseline camera on a small motorboat.

**Figure 12:**
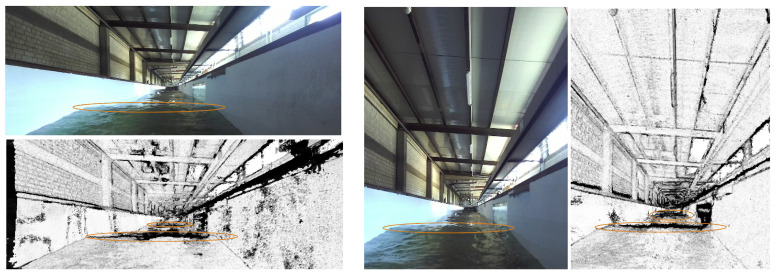
Low-angle confidence maps from an artificial wave flume. **Left**: horizontal setup. **Right**: vertical setup.

The second test took place in an artificial wave flume (supported by https://www.cameri-eng.com/ (accessed on 5 April 2023) CAMERI—Coastal and Marine Engineering Research Institute, Haifa, Israel). The flume has a piston-type wavemaker with an electric drive system and is equipped with an Active Wave Absorption Control System. We used a *ZED*2i stereo camera by StereoLABS (12 cm baseline) in both vertical and horizontal setups mounted on a 100 × 20 cm motorboat to record the artificial waves. The center of the stereo system was located ∼20 cm above the average water surface to achieve a low shooting angle. The artificial wave amplitude was set from 10 cm up to 20 cm, while the period was set from 3–7 s. Representative confidence maps, produced from vertical and from horizontal setup recordings from the flume, are presented in Figure 12. Note: The illustrations are presented on the basis of the confidence map since its high contrast makes it visually more adjusted to the human eye compared to the disparity map. In practice, the wave detection and tracking procedures were applied on the disparity map, whose “cleanliness” and smoothness are well-fitted to the suggested perception tasks, as is discussed in detail below.

Both the vertical and the horizontal setups did well isolating the occlusions caused by waves using the low confidence value they created on the confidence map. Yet it was visible that the vertical setup’s product ws sharper and cleaner than the horizontal setup’s product (in particular at long range), confirming our assumption following the typical horizontal structure of the waves. Another important outcome from this test is that using the stereo product as input for wave detection “exposed” waves not visible to the human eye using a mono RGB image (the farther ovals in both the vertical and horizontal setup; the vertical setup did better isolating the farther waves). This observation supports using stereo products over training DL model on mono RGB datasets for wave-detecting tasks.

Although the results from the flume experiment were encouraging, we wanted to test our methodology over real-world non-artificial data. The artificial waves generated by the mechanical wave generator in the flume did not contain any wind effects, which are common and crucial in real ocean wave structures. This fact—amongst other factors such as the artificial lighting in the flume that does not simulate real lighting conditions, the lack of chaos in the movement of the water surface, the monotony of the wave motion, and more—made us realize that a “real world” experiment was necessary. We equipped our motorboat with a Flight Controller (pixhawk 2.1) including IMU (9DoF) three MEMS-Gyros, three acceleration sensors, and three magnetic field sensors. Correlating the ship’s changing orientation with the observed wave may be useful for self-evaluation, and we went back to the open sea. All measurements, synchronizations, and computations were performed using a Jetson Xaviar nx computing platform (by NVIDIA, jetpack version: 4.5) in real-time and stored on a local hard drive. The visual products were transmitted to a remote monitor over WiFi. All implementation was performed with the assistance of StereoLABS’s dedicated Python API (pyzed.sl).

**Figure 13:**
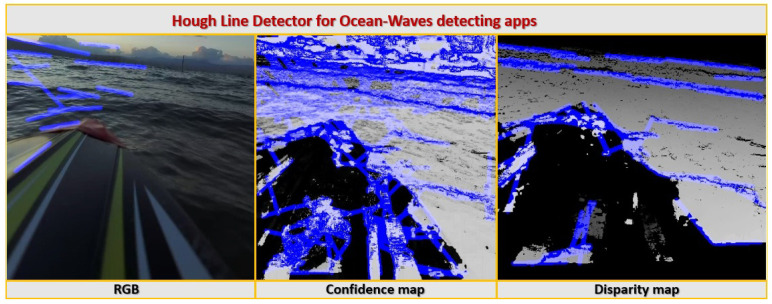
Hough line detector for ocean-wave detection from stereoscopic products from a vertical pair of ocean surface images.

Figure 13 compares the performance of a real-time Hough line detector on an RGB image of the sea surface, and on the corresponding confidence and disparity maps produced in real-time. The difficulty the algorithm has in recognizing sea waves from the RGB image is clearly noticeable. Operating on the RGB image, the algorithm is easily distracted and was unable to detect the lines representing sea waves. The products of the algorithm operating on the confidence map are a little surprising. On the one hand, there is significant improvement in the product of the algorithm operating on the confidence map compared to the one operating on the RGB image in terms of the detection of the waves over various ranges. On the other hand, the confidence map seems to be too “noisy” in a way that causes the algorithm to have many “False-Positive” errors—or false detection of lines that do not represent sea waves in reality. That is, it is difficult to extract the “real waves” out of all the false waves stemming from operating the line detector on the noisy confidence map. Operating the algorithm on the disparity map produced the desired results. Due to the dense mapping, the majority of the sea surface environment is “flattened” in the sense that reflections, refraction, and small waves do not get much expression in this map, but “significant” waves create occlusions that are expressed through “black holes” in the map. These “black holes” are very easily detected by the Hough algorithm, as shown in Figure 13. It can also be observed that the direction of deployment of the waves in a deep-water environment is approximately uniform. This observation can be used to efficiently and immediately filter out detection errors.

**Figure 14:**
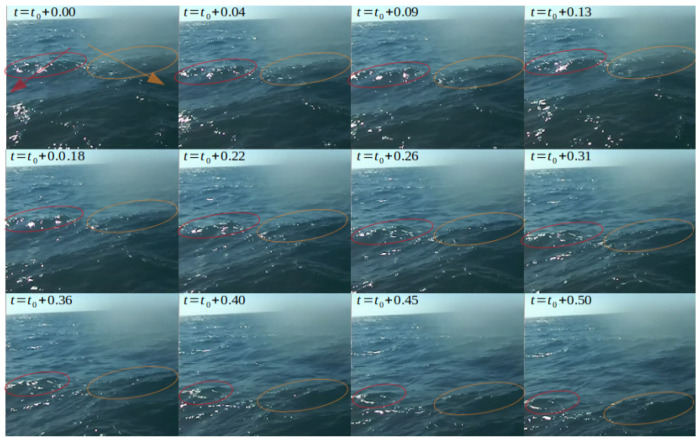
Two waves’ (marked with red and orange ovals) progress in 0.5 s. All time values are in s.

**Figure 15:**
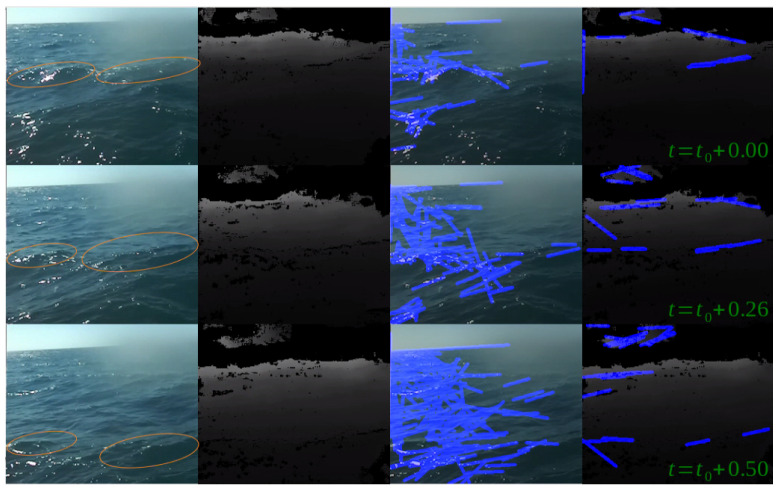
Hough-based wave detection in RGB and depth domains. All time values are in s.

Further comparison of Hough-based wave tracking between RGB and depth maps are presented in Figure 14 and Figure 15, where Figure 14 illustrates the progress of two waves over 0.5 s of video-capture in the RGB domain, and Figure 15 compares the performance of a simple off-the-shelf Hough-based wave detector and tracker in the RGB and depth domains over three consecutive frames (same scene as Figure 14) with a time-gap of 0.25 s.

**Figure 16:**
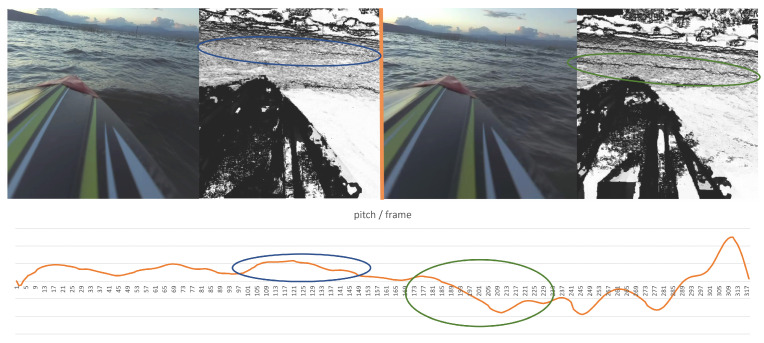
Two consecutive waves detected using the stereo product, and the corresponding recorded pitch motion. The second wave caused more significant pitch movement than the first one.

Once again, besides the success in wave detection, an important outcome stems from comparing the algorithm’s performance to human performance. While the flume experiment provided evidence that wave detection using a stereo product was better than human-eye-based wave detection over RGB images, the open-sea experiment suggested that wave characterization can be also improved using stereo products. As part of the experiment, the boat’s orientation was measured in parallel with the stereo recording. Hence, in addition to wave detection, we could also examine the boat’s movement in the presence of these waves. Those additional data enabled us to estimate each wave’s impact on the boat. Given that the wave’s occlusion causes a “black hole” in the stereo product, comparing the “hole’s” size between different waves may indicate the relative size of each wave with respect to other waves. In this way, the system can mark certain waves as “large” ones. This classification can be verified using the orientation measurements under the assumption that larger waves probably cause more significant impact to the boat. Figure 16 presents an example of two consecutive waves and their impact on the pitch channel of the ship’s orientation (marked with blue and green ovals, respectively). The two waves were detected at (approximately) the same distance from the boat. While the first wave (marked blue) was a “normal” wave, the second one (marked green) was large. The different size of the two waves is well-reflected in the pitch reaction.

Looking at the RGB images alone, it is difficult to determine which of the two waves is bigger (with a slight tendency to choose the first one, in the author’s opinion). Yet a look at the confidence maps shows unequivocally that the second wave caused a much larger occlusion, and hence it was bigger than the first one. This observation can form the basis for a future forecasting model of a ship’s 6DoF state in a wavy environment for various applications as described above.

## 5. Conclusions

The presented results confirm that the suggested approach enables low-cost yet efficient real-time ocean-wave detection and tracking independently performed by a small floating object using the disparity and the confidence maps generated by a stereoscopic imaging system rather than using the final 3D point-cloud only. It was also shown that the use of the disparity map outperforms human visual ability both in terms of wave detection and wave relative height classification according to their amplitude (e.g., “small”, “medium”, or “high” waves).

## 6. Discussion and Future Work

Projecting from the world of autonomous (off-road) vehicles, it seems that sensing the geometric surroundings of a robot seems to enable a large set of capabilities. Unfortunately, classical commonly used algorithms such as optical-flow, structure-from-motion, and SLAM have difficulty functioning in a marine environment. These algorithms count on a hidden assumption that some objects and feature points keep a stable structure between consecutive frames (in other words, at least a portion of the scene is static). As demonstrated in Figure 6, this assumption does not hold in the marine environment, and therefore, we suggest our model as a fundamental methodology for sensing the geometry surrounding a small floating object in a marine environment. As future work, we would like to start by creating a real-world benchmark of the ocean surface (in time); such a benchmark can serve as a basis for a wide range of AI problems. Moreover, we would like to use this benchmark in order to train a GAN (Generative Adversarial Network) framework in order to perform a simulated ocean surface that is as realistic as possible (in terms of geometry). Finally, there is a large space to improve the suggested stereo-based mapping method in terms of transforming the concept into an actual well-engineered model. The use of multi-cameras should have a more robust implementation (e.g., a rigid “L” shape with three cameras with a one-meter baseline). The use of IR projectors may allow the suggested method to perform well in low-light conditions. Finally, fusing (sensor fusion) wave radar with the suggested stereo vision solution may allow robust mapping in low-visibility conditions.

Being able to map the ocean surface in real-time using cost-effective commercial sensors (stereo vision) can lead to several new applications. Much like stereo sensors on commercial drones, which allow obstacle avoidance, 3D mapping, and real-time path planning, autonomous boats may be able to perform real-time control in order to minimize the boat’s rotation or optimize its energy consumption with respect to some required route. The computed ocean surface can be used as an input channel for a wide range of ML (Machine Learning) problems. We conjecture that such geometric data are highly suitable for a wide range of machine-learning problems, allowing a significantly faster (and better) learning curve with respect to vision-based input.

## Data Availability

Not applicable.
